# Individualized medicine enabled by genomics in Saudi Arabia

**DOI:** 10.1186/1755-8794-8-S1-S3

**Published:** 2015-01-15

**Authors:** Muhammad Abu-Elmagd, Mourad Assidi, Hans-Juergen Schulten, Ashraf Dallol, Peter Natesan Pushparaj, Farid Ahmed, Stephen W Scherer, Mohammed Al-Qahtani

**Affiliations:** 1 Centre of Excellence in Genomic Medicine Research (CEGMR), King Abdulaziz University, P.O. Box: 80216 Jeddah 21589, KSA; 2 KACST Technology Innovation Centre in Personalized Medicine at King Abdulaziz University (CIPM), P.O. Box: 80216 Jeddah 21589, KSA; 3 School of Biological Sciences, University of East Anglia, Norwich, Norfolk, NR4 7TJ, UK; 4 Zoology Department, Faculty of Science, Minia University, Minia, P.O. Box 61519, Egypt; 5 The Centre for Applied Genomics and Program in Genetics and Genome Biology, the Hospital for Sick Children, Toronto, Ontario, Canada; 6 McLaughlin Centre and Department of Molecular Genetics, University of Toronto, Toronto, Ontario, Canada

## Abstract

The biomedical research sector in Saudi Arabia has recently received special attention from the government, which is currently supporting research aimed at improving the understanding and treatment of common diseases afflicting Saudi Arabian society. To build capacity for research and training, a number of centres of excellence were established in different areas of the country. Among these, is the Centre of Excellence in Genomic Medicine Research (CEGMR) at King Abdulaziz University, Jeddah, with its internationally ranked and highly productive team performing translational research in the area of individualized medicine. Here, we present a panorama of the recent trends in different areas of biomedical research in Saudi Arabia drawing from our vision of where genomics will have maximal impact in the Kingdom of Saudi Arabia. We describe advances in a number of research areas including; congenital malformations, infertility, consanguinity and pre-implantation genetic diagnosis, cancer and genomic classifications in Saudi Arabia, epigenetic explanations of idiopathic disease, and pharmacogenomics and personalized medicine. We conclude that CEGMR will continue to play a pivotal role in advances in the field of genomics and research in this area is facing a number of challenges including generating high quality control data from Saudi population and policies for using these data need to comply with the international set up.

## Introduction

**Figure 1:**
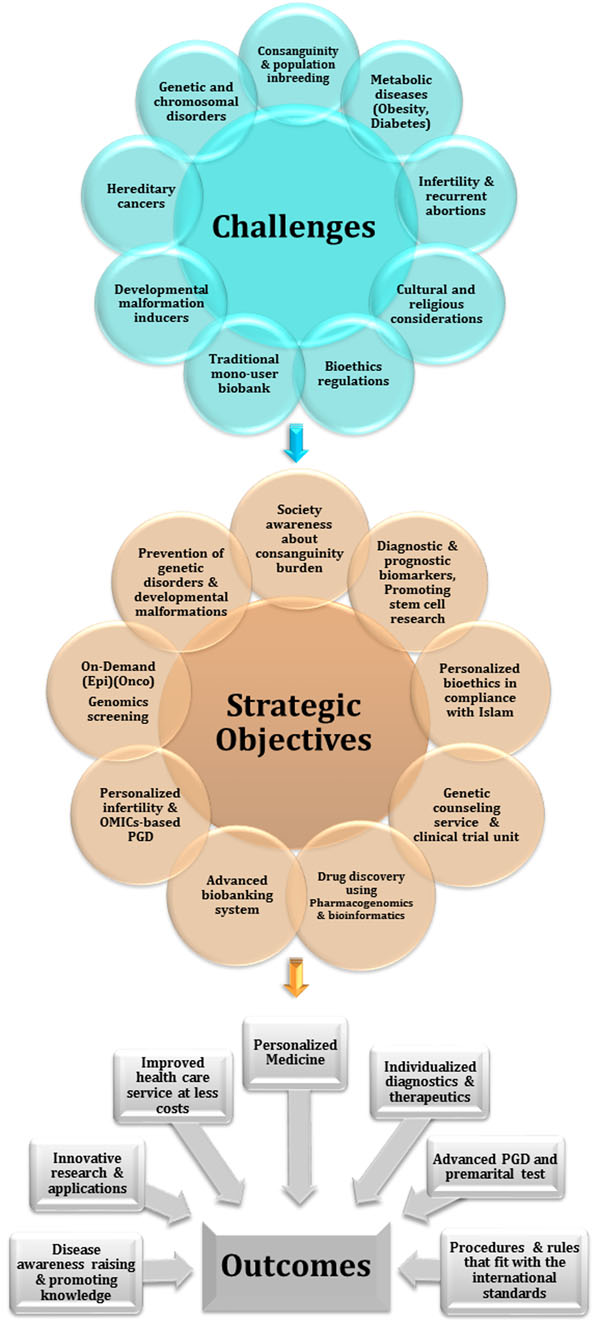
Challenges, objectives and expected outcomes of biomedical research in Saudi Arabia.

Leadership of the Kingdom of Saudi Arabia, is more conscious than ever of the necessity of establishing dedicated and long-term strategies that focus on the prevention of the effects of severe genetic disorders (most often due to consanguinity) and the impact of environmental changes (e.g., Dietary changes, toxins) on the general health of the Saudi society. As outlined in Figure 1, health policy planners and biomedical research scientists have conducted landscape surveys of the challenges facing the Saudi population, and formulated strategic objectives of how to move the health care research agenda forward with defined outcomes. Many of the decisions in investment in biomedical solutions for looming medical challenges are being grounded in establishing high-technology genomics and informatics-based centres that exploit the unique clinical and human resources available in the Middle Eastern countries, and in particular Saudi Arabia. International expertise is also being brought in through forward looking academic consultancies with universities and through new training initiatives.

In order to apply 21^st^ century solutions in biomedical health research, at least three foundational genomic research initiatives are under way in the Gulf Cooperation Council (GCC), which include Kingdom of Saudi Arabia, Qatar, Oman, Bahrain, Kuwait and United Arab Emirates. These initiatives include, the catalogue of transmission genetics in Arabs (CTGA) [1] hosted by the Centre for Arab Genomic Studies in the United Arab Emirates, the Qatar Biobank Initiative [2] and the Saudi human genome program (SHGP) [3].

Established in 2003, the main purpose of the CTGA is to increase public and academic awareness for early diagnosis of genetic diseases in the Pan Arab population and to translate scientific knowledge into individualized medical treatment and management programs. As of January 2014, the CTGA online database curates over 1,000 disease phenotypes with a focus on the epidemiology in the Arab world, and it also maintains a record of nearly 250 associated disease loci. Many of the disease phenotypes studied are either more frequent (such as G6PD deficiency) or are regionally endemic, restricted to certain tribal communities or even unique to consanguine families. Consanguinity, which is a main feature of the traditional Arabic culture (see below), leads to an increase of homozygosity for autosomal recessive genetic diseases which, from the perspective of research, can serve as an important repository for phenotype-genotype association studies [4]. The Qatar BioBank was initiated in 2010 aiming to take biosamples from Qatari population into repository to investigate diseases common to the domestic population [2]. Its ambitious goal is to serve as a driver for biomedical research inside and outside of Qatar. The SHGP intends to sequence the exomes and/or genomes of 100,000 Saudis within a period of five years taking advantage of the feasibility of next generation sequencing (NGS)[3]. In one of the large investments, NGS utilizing the Ion Proton technology (LifeTechnologies) has been allocated to the core facility of the SHGP in the capital Riyadh in addition to 15 planned satellite facilities across the Kingdom covering a substantial part of the 30 million nation’s populated areas. The satellites are established as collaborating institutions with self-adopted timelines and projects, which are led by on-site researchers independently from the SHGP headquarter.

Using the new genomic data, the SHGP is aiming to investigate a number of diseases, which are common or endemic in the country and have a strong genetic component such as diabetes, deafness, cardiovascular disease, neurodegeneration, cancer and inherited and Mendelian disorders (discussed below). Translational research aspects of this project include genetic counselling and in some instances further genetic testing of families where the underlying genetic mutations have been identified. For example, a number of genetic counselling services for inherited disorders like the pre-marital screening and genetic counselling program in Saudi Arabia for hemoglobinopathies and viral infections have been implemented [5]. Developing applications in pharmacogenomics research and personalized medicine are prospective objectives that may also arise from the SHGP. In addition, the aggregate genetic information offers the opportunity to perform genome wide association studies (GWAS) in a new dimensional scale. Such studies may shed light upon a number of polymorphic traits that are associated with certain diseases and are suspected to add to their penetrance which may gain special importance for endemic conditions like body mass index and obesity [6], or for acute diseases like cancer [7].

**Figure 2:**
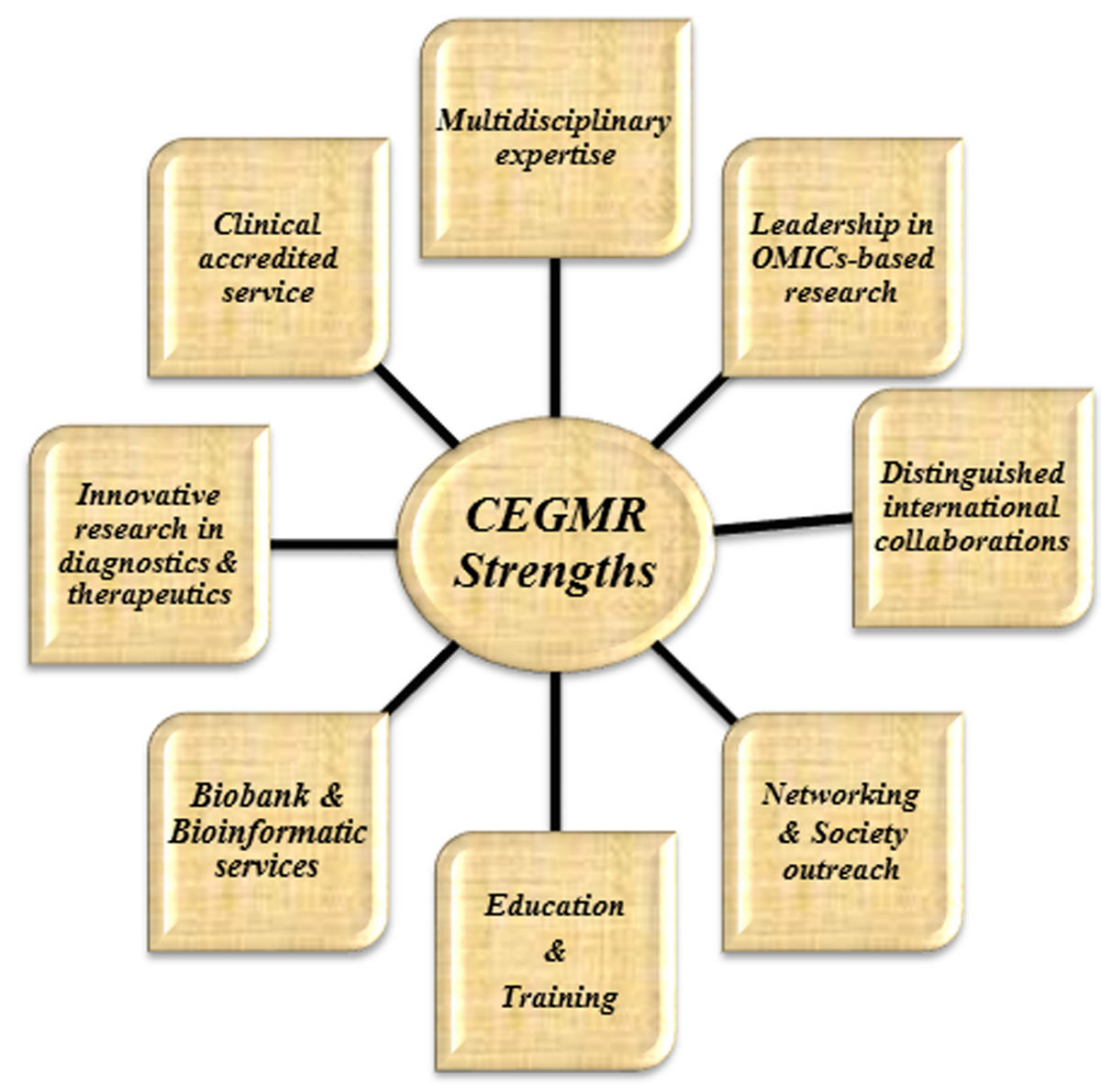
Centre of Excellence in Genomic Medicine Research (CEGMR) Multi-disciplinary platforms in biomedical research.

Our review is divided into five themes that we believe are most relevant for genomic technologies to have impact in Saudi Arabia. We ordered the themes based on our interpretation of the challenges unique to Saudi Arabia and the Middle East (Fig. 1). We believe the investment in research will have significant impact on our own health care systems and the lessons learned will also have an impact in other regions of the world, in particular as population migration expands and genetics becomes fully integrated into medical management for individuals and their family members [8]. Our vantage point is from working within the Centre of Excellence in Genomic Medicine Research (CEGMR), King Abdulaziz University (KAU), Jeddah, Kingdom of Saudi Arabia (KSA). This dynamic organization is aiming to develop and apply the best practices in genomic medicine driven by world-class research programs (Fig. 2) to further the health of citizens of Saudi Arabia and the world.

### I. Congenital malformations in Saudi Arabia

Congenital malformations are identified as physical irreversible alterations induced during embryo formation that can be grouped into five categories: (1) single-gene anomalies, (2) chromosomal abnormalities, (3) maternal illness conditions during early pregnancy, (4) environmental and genetic inducers (e.g., teratogens), and (5) unknown causes, which account for ~50% of congenital malformations [9].

**Figure 3:**
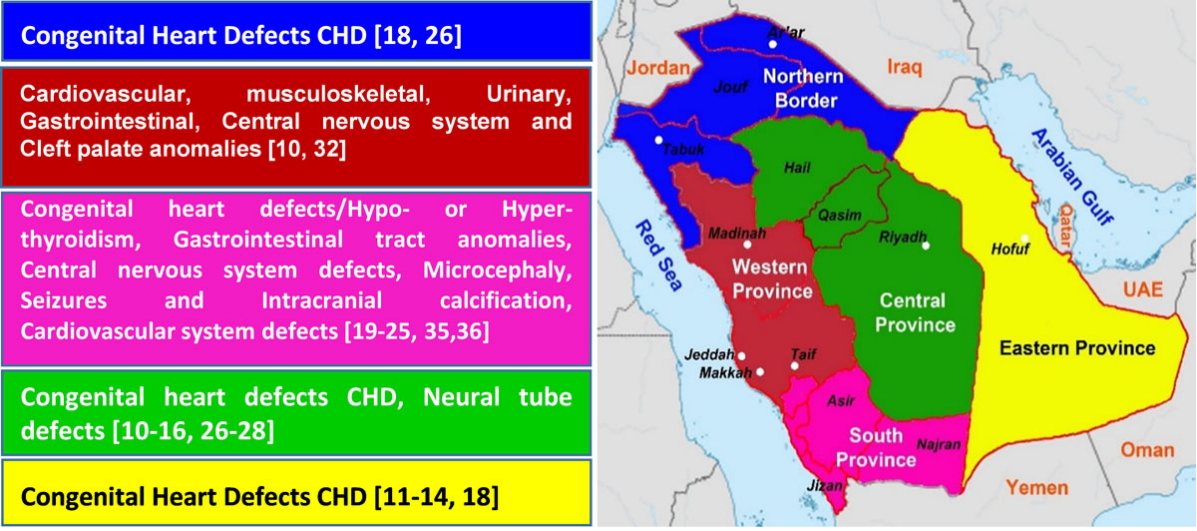
Regional prevalence of cardiac heart defects (CDH) in the Kingdom of Saudi Arabia.

In Saudi Arabia, a number of studies were carried out to screen for congenital abnormalities in different regions of the kingdom (Fig. 3). In the Western province of Saudi Arabia, a high prevalence of major congenital abnormalities including cardiovascular, musculoskeletal, urinary and central nervous system (CNS) was reported [10]. Interestingly, this study showed that at least 38% of the parents of babies with congenital malformations were consanguineous. More supporting evidence from large multicentre studies conducted across Riyadh and the Eastern region revealed that first cousin marriage have a direct link with a number of congenital heart defects (CHD) [11-14]. Severe CHD were also reported in large studies in Al-Qassim (central region) with authors attributing the severity to the high rate of consanguinity [15,16]. These findings are not surprising since Saudi Arabia has the highest consanguinity among all middle eastern countries [17]. Based on Saudi Arabian regional distribution, South-Western and Northern provinces have the highest prevalence of CHD (Fig. 3)[18]. In Najran in South province, a significant high prevalence of hypothyroidism and hyperthyroidism associated with CHD were reported and attributed to high rate of consanguinity [19-21]. In Asir region in the South province, a high prevalence of a number of congenital malformations was detected in newly born babies and found to be linked to high consanguinity. Among these are gastrointestinal tract anomalies [22], CNS defects [23,24] and cardiovascular system defects [25]. All previous studies used hospital database analysis, however, the first community based study to measure the prevalence of CHD was carried out by Alqurashi, et al., 2007 [26]. This study showed that the central region had the highest CHD prevalence of 27 per 10,000 and the north-western was the least affected region with prevalence of 9 per 10 000 [26]. A wide range of the CNS malformations including neural tube defects have been detected in different regions of Saudi Arabia with consanguinity appearing to be a major risk factor [23,24,27,28]. The mountainous geography of Saudi Arabia may also contribute to the high rate of congenital malformations since high altitude has been reported in other regions worldwide as a cause of congenital anomalies [29-31]. In Al-Taif city, known as a very high-altitude area, an increase in cardiovascular, urogenital, musculoskeletal, gastrointestinal, and cleft palate anomalies in addition to chromosomal abnormalities were reported [32].

Due to strong economic stability, obesity in Gulf countries in general and in Saudi Arabia and Kuwait in particular has become a high risk factor and a serious problem that reflects negatively on the these societies [6]. According to the Saudi Arabian national nutrition survey issued in 2007, the prevalence of obesity among women is 23.6% compared to 14.2% in men [33]. It has been reported that a mother’s body mass index of more than 30 is a predictor of a number of congenital malformations including cardiac, nervous system, facial and genitourinary tract defects [34]. Environmental factors and metabolic disorders are believed to induce congenital infections with toxoplasmosis, and cytomegalovirus that in turn result in inducing a number of congenital malformations including; microcephaly, seizures and intracranial calcification [35,36].

Different genomic research approaches to elucidate the causes of congenital anomalies in Saudi Arabia have recently begun to gain more attention. For example, a multi-level technology approach was used to unravel the molecular network that lead to cryptorchidism [37]. Comparative genomic hybridization (CGH) was also used to analyse chromosome microdeletions that caused upper limbs disabilities [38], and exome sequencing led to the identification of the *ADAMTS18* gene to be involved in eye development [29].

Despite a recommendation to launch a national screening program for congenital anomalies in 1997 [20], there remains a real need for a primary national prevention program. We believe this type of program should be congruent with an awareness program of the genetic and epigenetic causes of the birth defects in Saudi Arabia. There is support for the implementation of an extensive pre-marital and pre-implantation screening program, in addition to establishing a national database for congenital anomalies, and by offering genetic counselling when birth defects occur [39,40]. Recently, King Faisal Specialist Hospital & Research Centre (KFSH & RC) in Riyadh launched a technical database core facility that aims to maintain database registries of a number of diseases and congenital defects in order to facilitate clinical and research domains in Saudi Arabia (http://rc.kfshrc.edu.sa/besc/sections/tdbcf/OurProjects.html). Among these registries are; new-born screening and biochemical genetics (http://nlnbs.kfshrc.edu.sa), cleft lip/palate and craniofacial anomalies (http://rc.kfshrc.edu.sa/bssc/clcpr_new/buttons/New_Site.htm), congenital heart defects (http://rc.kfshrc.edu.sa/chd_program/) and neural tube defects (http://rc.kfshrc.edu.sa/ntd/). Establishing similar regional initiatives connected to a central database facility would reveal the most accurate data on congenital malformations in Saudi Arabia.

At CEGMR, a number of research initiatives are already in place to screen and investigate the causes of congenital malformations in Saudi Arabia. Among these initiatives are KACST’s recently funded projects to apply microarray based CGH approach to unravel genome defects in the congenital heart diseases and another to screen and diagnose the developmental delay as well as other congenital malformations. In addition, CEGMR established a collaboration with the Environmental Sciences Department at KAU to analyse changes in gene expression profiling using array CGH, RNA-Seq and whole exome sequencing during early embryonic development that could be induced by air particulate pollution and pesticides.

### II. Infertility and pre-implantation genetic diagnosis (PGD)

Infertility is a condition manifested by the biological incapacity of a couple to achieve a clinical pregnancy after at least one year of regular unprotected sexual intercourse [41]. The prevalence of this disease continues to rise and affects about 1 out of 6 couples in Saudi Arabia and worldwide [42,43]. Such infertility rates vary largely among countries and patient age [44]. The most common factors that could explain this severe decline in fertility are reproductive aging through the postponement of marriages, lifestyle, nutrition, the quality of health care services, and decrease in the child-seeking behaviour [45-47]. These factors are mainly due to the maternal age and to a lesser extent the paternal factors that could correlate with increased rates of gametes’ chromosome cohesion and therefore higher aneuploidy and chromosomal abnormalities in blastocysts [48-50].

There is an increase in the number of couples seeking infertility treatment services in assisted reproductive technologies (ART) clinics to help them to procreate. Despite valuable ART advantages, a remaining challenge is to increase qualitatively and quantitatively the *in vitro* fertilization (IVF) outcomes and reduce health care costs for both the mother and the offspring [51,52]. The gametes’ (spermatozoa and oocyte) quality has been recognized as the major limiting step and is therefore the target of numerous studies to improve their selection procedure [53-56], in order to make the elective single embryo transfer (eSET) procedure more effective [57,58]. Chromosomal and some genetic mutations have been reported to underlie many of the challenges for obtaining successful embryo implantation and subsequent delivery of healthy babies [59-61].

In addition to the infertility challenges, Saudi society in particular is characterized as mentioned above by an extremely high-level of consanguinity. In fact, large proportions of marriages are still driven by ethnic or tribal considerations. The rate of consanguineous marriages is about 58% amongst them 60% were between first-degree cousins [12,17], which represents one of the highest in the Middle East and worldwide. In addition to the increase of the frequency of inherited recessive disorders, these consanguineous marriages have direct effects on some reproductive health parameters such as infertility rates, recurrent abortions, congenital disorders and neonatal death (Table 1) [4]. It should be highlighted that these genetic and chromosomal disorders’ rates are variable among regions inside Saudi Arabia. Additionally, these rates are exceptionally high in Saudi Arabia and Middle East in general compared to other regions in the world due to the burden of consanguinity and population inbreeding.

**Table 1:** An overview of the main genetic and chromosomal disorder in Saudi Arabia and their relationship with consanguinity. (++): High correlation; (+): Positive correlation; ND: Not Determined.

Type	Disease	Disease prevalence* (%)	Consanguinity	References
**Chromosomal**	Down syndrome	1.8	ND	[172]
	
	Congenital Malformations or birth defects	2- 3	++	[12,13,173]

**Polygenic and/or multifactorial**	Congenital heart disease (CHD)	5.4-10.7	+	[15,174,175]
	
	Cystic fibrosis	0.24	++	[176]
	
	Duchene muscular dystrophy (DMD)	0.025	+	[177]
	
	Hereditary Recessive deafness	130	++	[178-180]
	
	Hereditary blindness & visual impairment	90	++	[181,182]
	
	Diabetes mellitus	110 to 237	ND	[183,184]
	
	Thalassemia	0.5 to 2.6	++	[185-188]

**Monogenic**	Sickle Cell Disease (SCD)	9 to 14.5	++	[185,187,189-192]
	
	G6PD deficiency	7.7 to 20	++	[12,193,194]

*Rates from national and/or regional studies done in Saudi Arabia

Given the challenges of high rates of consanguinity and disease in the Middle East and in Saudi Arabia, the Saudi ministry of health (MOH) decided in 2004 to make the premarital testing compulsory [5,62]. Such premarital examination includes mainly the basic blood tests, the hemoglobinopathies screening (Thalassemia & Sickle Cell Anaemia) and virology assays (Hepatitis B and C; AIDS). Despite these important measurements, PGD is still restricted to only few IVF clinics and it is the opinion of the authors that the level of awareness about infertility problems and genetic diseases needs to be given more attention especially in regions with high consanguinity as well as in rural and tribal areas. Investment in training and staffing in genetic counselling will also then be required.

The CEGMR aims to play a leading role in PGD by building translational research capacity. CEGMR is currently working in collaboration with KACST Centre of Innovation for Personalized Medicine (CIPM), Jeddah at KAU; the latter is being involved in delivering service through PGD screening for the larger Saudi population. CEGMR is currently developing new infertility screening methods using a specific panel of biomarkers with the primary aim being to reduce the comparatively high recurrence of abortions. For examples, new advances in technology have allowed the transfer of two embryos to the eSET preventing multiple pregnancies, which can disadvantage both the mother and the baby [63]. Similarly, a new project was recently initiated to establish an advanced premarital screening (APS) using state-of-the-art approaches with higher coverage (number of diseases) and accuracy. Once validated, such APS package will be presented to the MOH for consideration as an improvement of the existing test using advanced high throughput technologies.

Together, fertility and PGD screening programs as well as the APS package are excellent tools that are on line with the latest advances in the reproductive and developmental medicine field worldwide and are expected to significantly enhance the efficiency of both infertility diagnosis and treatment [64-66]. They will also facilitate detection and prevention of the transfer of most of the inherited disorders to the upcoming generations.

### III. Cancer and genomic classifications in Saudi Arabia

**Figure 4:**
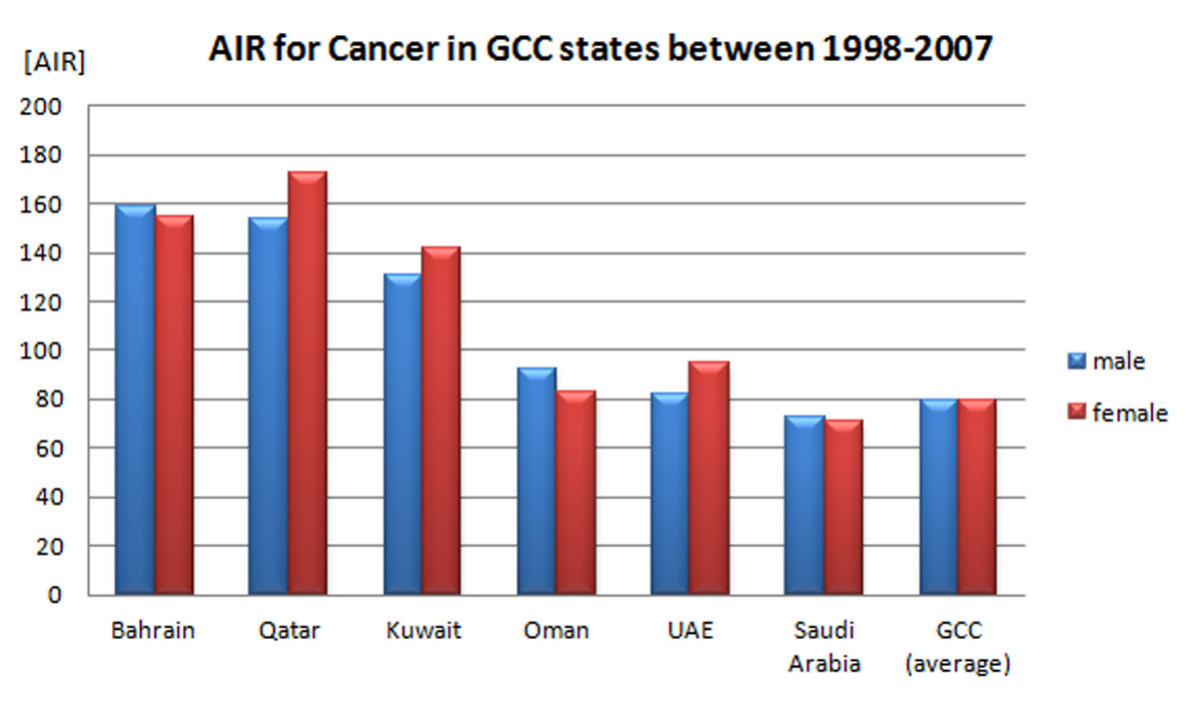
Average annual age standardized incidence rates (ASR) of all cancers in males and females from the GCC states in the period 1998-2007 [68]. For comparison, ASR for cancer in the US in the period 2005-2009 ranged between nearly 290 in Asian American and Pacific Islander women and approximately 620 in African American men [91].

All six GCC countries curate national cancer registries that provide statistics measures to the Gulf centre for cancer registration [67-70]. Remarkably, the national cancer registers of Bahrain, Kuwait, Oman, and Qatar are listed in a high incidence data quality category as assessed by the international agency for research on cancer (IARC) [71]. In comparison with USA, cancer data indicates a lower incidence rate for cancer in the Gulf region (Fig. 4); yet, a certain deviation of the frequency of the most common cancer types is observed [72]. For example, thyroid and liver cancer, as well as leukaemia and non-Hodgkin`s lymphoma (NHL) are relatively more common than in the USA (all races) [68,72]. Thyroid cancer is the second most frequent cancer in young women in the majority of the GCC countries [73,74]. Contrarily, prostate cancer is less frequent in the Gulf region, especially in Saudi Arabia [75]. In most common cancer types including thyroid and breast cancer, patients are several years younger at first diagnosis compared to Western countries [67,72,76,77]. Molecular genetics studies from the Gulf region have demonstrated that hereditary components like single nucleotide polymorphisms (SNPs) confer risk for breast [78] and thyroid cancer [79,80]. More specifically for the p53 codon, 72 polymorphism showed an association with early onset of breast cancer in Saudi women [81].

**Figure 5:**
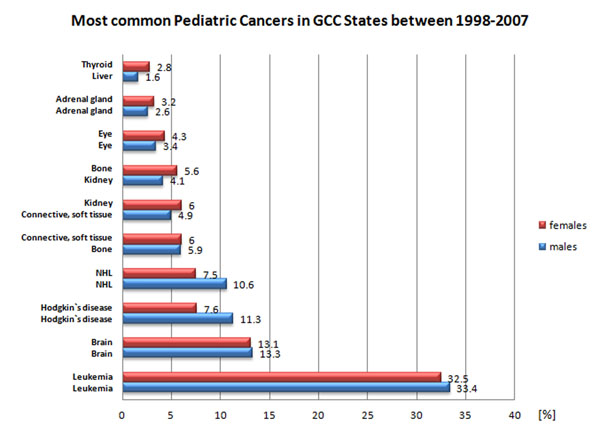
Most common cancer types in the period 1998-2007 for children between 0-14 years from the GCC states [68]. Leukaemia, brain cancer, Hodgkin’s diseases, and NHL were the most common malignancies in both genders.

A high prevalence of childhood cancer in relation to adulthood cancer is observed in the GCC region. This discrepancy is in first instance attributed to the young population structure of the region [67,82]. Similar to Western countries, leukaemia and lymphomas including both Hodgkin's lymphoma and NHL are among the most frequent paediatric cancers (Fig. 5). To provide specialized paediatric cancer care, the King Fahd National Centre for Children's Cancer and Research, hosted by KFSH & RC, was established in 1997 in the Saudi capital Riyadh. The paediatric clinic, the largest in the region, performs approximately 70 stem cell transplantations per annum. One of the research areas of both institutions is focusing on the molecular genetics of diffuse B-cell lymphomas, which are the most common subtype of extranodal NHL and occur with a comparably high prevalence in the Gulf region [83-85]. Notable findings include detection of clinically relevant biomarkers such as* SKP2*, *XAIP*, *FOXM1*, and *MET* in diffuse large B-cell lymphomas and detection of molecular mechanisms for *NFĸB* in activated B-cell lymphomas [83,86-89]. For acute lymphoblastic leukaemia (ALL), a Saudi retrospective multicentre study revealed clinical characteristics similar to those found in developed countries; however, the authors noticed that standardization of patients` care has to be improved [82]. Interestingly, an epidemiological survey in the Detroit area of USA revealed a higher incidence of leukaemia and bladder cancer in US-born males of Arab origin compared to non-Hispanic whites [90] indicating a possible involvement of inherited susceptibility factors in these malignancy types, as shown for other diseases like thyroid and breast cancer [79-81,91]. These findings emphasize the genetic heterogeneity of major cancer types, in which NGS may become the tool for personalized medicine intervention.

### IV. Epigenetic explanations of idiopathic disease

A marked feature of cancer in the Kingdom of Saudi Arabia is the relatively young age of onset. The most common cancer type in Saudi Arabia is breast cancer comprising more than 16% of the cases seen at the largest oncology referral centre in the Kingdom, KFSH & RC [92]. Most of the affected females are below 50 years of age [92], which differs from Western countries where the age of onset is later [93]. Colon cancer is the most cancer type affecting Saudi males and here too the age of onset (40-65 years) [92] is less than seen in the west [93]. The reason for such age discrepancy is not entirely known. However, factors such as the early age of menarche (<12 years) and the use of exogenous progesterone and oestrogens could be suspected in the case of breast cancer [94]. The relatively young age of onset in this population could be explained by the interplay between common genetic susceptibility background substantiated by increased consanguinity and epigenetic aberrations. These factors are thought to be caused by shifts in life style being experienced in Saudi Arabia and in the Middle East in general in the past few decades. Similar life-style shifts are also seen in emerging economies such as India. Similar to Saudi Arabia, there is an increasing number of younger patients diagnosed with cancer every year in India [95]. The presence of a common genetic susceptibility factor(s) amongst Saudis is unknown. Interestingly, no significant associations of BRCA1 and BRCA2 mutations or SNP have been found in Saudi breast cancer cohorts [96,97]. However, there is an ongoing study to elucidate this further at CEGMR using high throughput technologies including Ion Torrent whole genome sequencing. In addition, our exome sequencing efforts of familial breast cancer cases have yet to reveal a common gene or mutation in the samples so far analysed [98]. Interestingly, every family sequenced thus far has a different underlying putative defective gene(s) precluding our ability to provide supporting evidence for the hypothesis that suggests the presence of a consanguinity-driven common genetic haplotype associated with increased susceptibility to breast cancer [98]. A more defined role for a “cancer susceptibility genome” will be determined following the conclusion of the SHGP project [99].

Epigenetics could therefore play a major role in the shift towards a younger age of onset. Life-style changes such as sleep and shift work patterns, diet and exercise can affect the genome in several ways and need to be considered. The circadian rhythm, mediated through an intricate network of transcriptional and post-translational mediators is necessary for maintaining a healthy homeostasis [100-102]. When the circadian rhythm is disrupted through sleep deprivation, global changes in gene expression can occur altering cellular behaviour. Methylation is a key mechanism for altering gene expression in response to sleep deprivation, such as what happens to the brain function [103]. The pleiotropic melatonin is the main sensor for light and it has cancer-protective effects through several mechanisms, including regulating estrogen pathway [104]. Interestingly, we have identified frequent hypermethylation of the melatonin receptor, *MTNR1B*, in a fraction of our cohort of breast cancer cases (35%) with significant association with histological grade I [105]. Such a result is intriguing and could implicate the epigenetic deregulation of key components of the circadian clock, which in turn, could potentiate cancer at an early age [100,101].

Sleep deprivation is not the only suspect behind the age shift observed. Aberrant metabolism is also likely to play a role. The incidence rates of the metabolic syndrome (obesity, high blood pressure, elevated fasting glucose levels, elevated cholesterol levels) in Saudi Arabia are reported to be among the highest in the world [106]. There is also emerging evidence of metabolic syndromes contributing to the increasing rates and age shift of cancer in Saudi Arabia. Our genome-wide methylation analysis of breast cancer has identified a role for the *KLOTHO-FGF19-FGF23-FGFR4 *signaling axis. FGF19 expression is elevated and is associated with a significantly shorter life expectancy of breast cancer patients of Saudi and Libyan descent [107]. The upregulation of *FGF19 *expression is coupled with high FGF23 and FGFR4 expression and frequent methylation of KLOTHO in the same cohort (Unpublished data). While a role for FGFR4 has been previously shown [108,109], implicating the metabolic hormones *FGF19*, *FGF23 and KLOTHO* in cancer is equally intriguing; the emerging question being if dysregulation of these genes may be a direct cause of carcinogenesis or more generally a symptom of metabolic health, which in itself predisposes to cancer at a significantly younger age? More work is warranted to shed light on the role of this metabolic axis in cancer because of its potential as an actionable target for treatment [110,111].

Other genes are inactivated by promoter hypermethylation. In colorectal cancer from Saudi Arabia, the methylation of the polycomb gene targets in the absence of CpG island methylator phenotype (CIMP) is a predictor of favourable prognosis [112]. Interestingly, we have identified a group of older patients with no detectable methylation in the genes analysed and yet displayed aggressive tumours that led to a significantly lower disease-specific survival rates [112]. The most frequent methylation event detected in breast cancer from Saudi cases is at the *RASSF1A* promoter, an event that is a potential predictor of poor prognosis [113]. However, the methylation frequency of other genes analysed in that study was lower than reported elsewhere, especially in the case of Estrogen Receptor-1 (ESR1) and Cadherin-1 (CDH1) methylation [113]. This could be attributed to the relatively early age of onset for the patients analysed, or to the presence of regional and ethnic-specific differences in gene regulation in breast cancer. BRCA1 methylation in blood from breast cancer patients could provide a potential mechanism for cancer susceptibility in the Saudi population and offer a promising tool for the detection of cancer predisposition [114]. Amplification of TNRC9, a common event in breast cancer from the MENA region, can directly result in the epigenetic silencing of BRCA1 expression [115]. Incidentally, SNPs in the TNRC9 locus have been shown to be significantly associated with breast cancer [116].

Other epigenetic lesions underlying cancer in Saudi Arabia could exist. Differential expression of miRNAs including hsa-mir-146a2, hsa-mir-196a2 and hsa-mir-499 are detected in peripheral blood of breast cancer patients [117]. Furthermore, polymorphisms identified in those miRNAs have been found to be significantly associated with breast cancer susceptibility [117]. A similar effect is observed in risk modification for prostate cancer in northern Indian populations [118]. Polymorphisms in hsa-mir-196a has been shown to be associated with increased risk for nasopharyngeal carcinoma in Chinese patients [119], and with increased risk for non-small cell lung carcinoma in the Korean population [120]. Conversely, these genetic variants do not seem to influence breast cancer risk or age of onset in German or Italian populations [121].

### V. Pharmacogenomics and personalized medicine

Pharmacogenomics (PGx) is the study of the role of genetic polymorphisms in drug response [122,123]. PGx is a rapidly evolving field in particular due to the rapid advances in genotyping platforms and NGS technologies to scan the genome at increasing levels of resolution [124]. The interface of PGx and complex diseases including various types of cancers, understanding and managing serious adverse drug reactions (SADRs), and public health pharmacogenetics, are the major areas of investigation at CEGMR and elsewhere [124,125].

The potential opportunities emerging from PGx include, but not limited to, the prediction of personalized response to drugs, tailor-made prescriptions, reducing adverse drug reactions (ADRs), increasing the efficacy and reducing the toxicity of drugs, developing more robust and safer vaccines, monitoring and screening of specific diseases, as well as advancing research and development of novel drugs [122]. Pharmacogenetics (PGt) is the study of single genes that have significant influence on clinically actionable drugs [126].

In the past two decades, several drugs were primarily withdrawn from the USA market due to ADRs [125,127,128]. The ADRs are broadly categorized into Type A and B reactions [127]. Type A reactions are predictable, dose-dependent and constitute about 80% of ADRs [125,127]. The hypotension with anti-hypertensive treatment and incidences of bleeding in patients treated with Warfarin are some of the examples of Type A ADRs [125,127]. However, Type B reactions are termed as “idiosyncratic” or random ADRs observed mostly in genetically susceptible individuals [127]. Moreover, Type B reactions may potentially lead to SADRs that require either temporary or prolonged hospitalization and in some instances permanent disability or are even fatal [125,129]. Deciphering the genetic variations in populations [130], associated with Type B reactions using PGx strategies, could markedly reduce the healthcare budgets, promote drug discovery and the development of reliable and safer drugs [125,129].

The Cytochrome P450 (*CYP450*) super-family consists of functionally diverse groups of drug detoxifying enzymes encoded by 57 genes and 5 pseudo genes, identified thus far, in the human genome [131,132]. The CYP450 enzymes are chiefly expressed in liver and facilitate the metabolism of drugs, xenobiotics, toxic pollutants, steroids, vitamin D, cholesterol metabolism and bile synthesis, retinoic acid, arachidonic acid and eicosanoids [132,133] by conjugating with functional groups and eventually removal from the body [132,134,135]. Members of the CYP450 super-family like CYP1, CYP2, and CYP3 are essential for the phase I metabolism of about 90% of all clinically applicable drugs [134-136]. The personalized variations in the expression of chief detoxifying CYP450 enzyme systems in humans, such as *CYP1A2*, *CYP2C*, *CYP2D6*, *CYP2E1*, *CYP3A4* and *CYP3A5*, can influence the metabolism of a clinically relevant drug and the ADRs associated with its standard doses [124,135,136]. An array of factors such as the genetic polymorphisms, poor diet, endocrine dysregulation, epigenetic changes, acute and chronic liver diseases can critically influence the personalized variation in the expression of CYP450 system [135,137]. Besides, the CYP450 clinical phenotypes are classified based on individuals’ ability to metabolize a particular drug as ultra-rapid metabolizers (UMs), Extensive Metabolizers (EMs), Intermediate Metabolizers (IMs) and poor metabolizers (PMs) [138,139]. The PMs have two non-functional alleles that decrease the expression of a particular CYP450 enzyme [136]; IMs have one functional allele and one heterozygous null allele for a CYP450 enzyme [136]. EMs have two functional homozygous alleles and may require higher concentrations of a therapeutic drug compared with IMs and PMs [136]. UMs have two or more copies of a functional CYP450 gene and metabolize the drug substrates rapidly and always require higher concentrations of drugs to achieve good therapeutic outcomes [136].

Genetic polymorphisms of CYP450 superfamily of genes greatly influence the efficacy, safety and the clinical outcome of a drug treatment [136]. *CYP2D6* is one of most important CYP450 enzyme required for the metabolism of more than 30% of therapeutically relevant drugs, including antidepressants and antipsychotics [138,139]. It is polymorphically expressed, and found to be absent in 1- 2% of Asians, 6 - 10% of Caucasians [139] and are classified as PMs [138,139]. Over 25% of Ethiopians and 2% of Caucasians are classified as *CYP2D6* UMs [138,140]. In general, PMs of drugs have an increased risk of developing ADRs when compared to UMs and EMs. For example, codeine, a pro-drug, is converted to morphine by CYP2D6 enzyme to exert its analgesic properties [140]. Therefore, the efficacy of codeine was significantly reduced in individuals with *CYP2D6* PMs [140]. On the other hand, the efficacy of codeine was markedly increased in *CYP2D6* IMs and *CYP2D6* UMs leading to drug toxicity in some individuals [140].

CYP2C subfamily of enzymes metabolizes about 20% of clinically relevant drugs including non-steroidal anti-inflammatory drugs (NSAIDs), oral anticoagulants and oral anti-diabetic agents such as tolbutamide, glibenclamide, glimepiride and glipizide [141-143]. To date, more than 57 alleles of CYP2C9 have been reported [144] amongst which CYP2C9*2 and CYP2C9*3 alleles are the most prevalent in Caucasian as well as Asian populations and individuals carrying these polymorphisms exhibit reduced CYP2C9 activity [141]. An association was established between reduced expression of CYP2C9 and higher risk of bleeding episodes and upper-gastrointestinal (GI) bleeding in Warfarin-treated patients [136,145]. The alleles of CYP2C9 namely, CYP2C9*2 and CYP2C9*3 are responsible for reduced CYP2C9 enzyme activity leading to increased anti-coagulation [136]. These patients should be administered with lower dose of Warfarin to attain effective anti-coagulation, and diminish the possibility of bleeding episodes [136,145,146].

**Figure 6:**
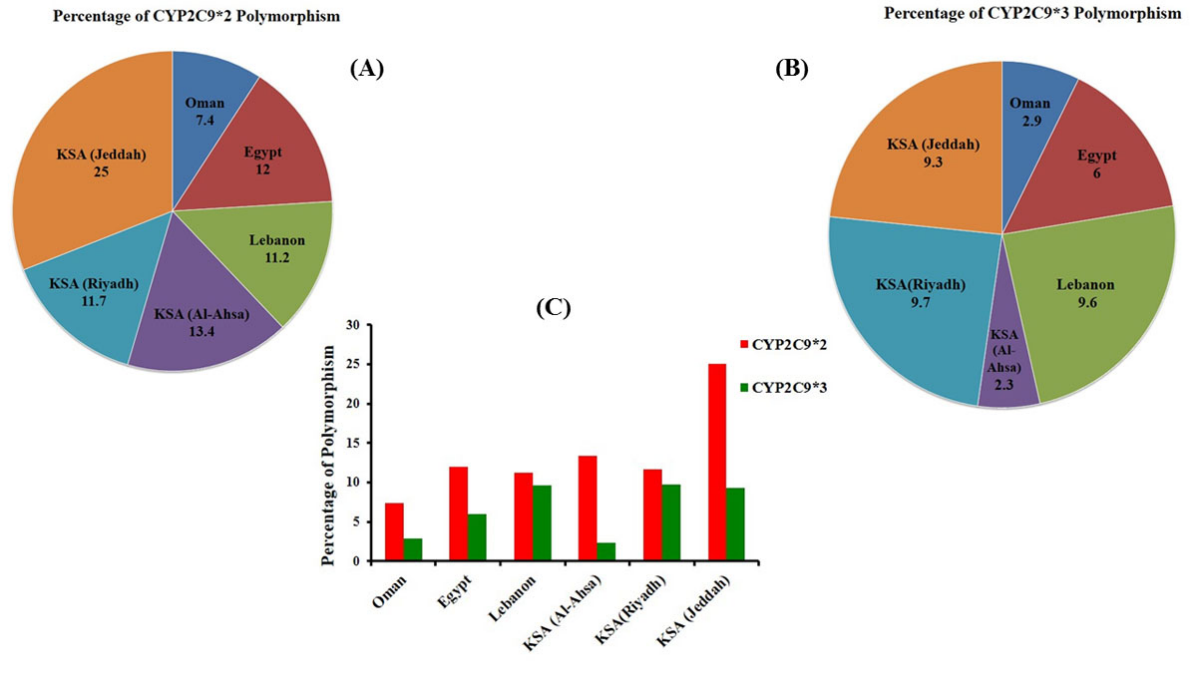
(A) Pie-chart represents the CYP2C9*2 and (B) CYP2C9*3 polymorphisms observed in some of the Middle Eastern countries. (C) The percentage of CYP2C9*2 and CYP2C9*3 polymorphisms identified in Oman, Egypt, Lebanon, as well as in Al-Ahsa, Riyadh and Jeddah regions of the KSA. The incidence of CYP2C9 polymorphisms in Saudi population was similar to Caucasians; but, it was more than Asians and Africans [169] and adverse bleeding episodes are not uncommon in Saudi patients with CYP2C9*2 and CYP2C*3 alleles and may require 40% less Warfarin dose to induce effective anti-coagulation [169].

In Oman, the frequencies for CYP2C9*2 and CYP2C9*3 alleles were shown as 7.4% and 2.9% respectively [147,148]. In Egypt, the allelic frequencies of CYP2C9*2 and CYP2C9*3 alleles were estimated as 12% and 6% respectively [149]. In Lebanon, the allele frequencies for these two alleles were found to be 11.2% and 9.6%, respectively [148,150]. The frequencies of CYP2C9*2 and CYP2C9*3 alleles among Saudis in Al-Ahsa were about 13.4% and 2.3%, respectively [148]. In Riyadh, the frequencies for CYP2C9*2 and CYP2C9*3 alleles were reported to be 11.7% and 9.7% respectively [143]. The analysis of common genetic variants of the CYP2C9 subfamily in type 2 Diabetes mellitus (T2DM) patients in Jeddah showed that the allele frequencies of CYP2C9*2 and CYP2C9*3 were 25% and 9.3%, respectively (personal communication, Nehad Abdulqader Shaer and Ashraf Dallol). It was recently shown that the incidence of CYP2C9 polymorphisms in Saudi population was comparable to Caucasians; however, it was higher than Africans and Asians [151]. Saudi patients with CYP2C9*2 and CYP2C*3 alleles had more adverse bleeding episodes and require 40% less Warfarin dose to cause effective anti-coagulation (Fig. 6) [151].

In December 2004, the United States food and drug administration (FDA) approved the drug metabolizing enzyme (DME) genotyping system from Roche named as AmpliChip^TM^CYP450 array [152]. The AmpliChip P450 array is used in Affymetrix GeneChip microarray instrumentation system to identify a patient’s CYP2D6 and CYP2C19 genotypes from genomic DNA isolated from whole blood sample [152]. Based on the CYP450 genetic test results, clinical evaluation and other lab tests as a reference, the clinician designs personalized treatment options for patients to minimize the risk of ADRs and sub-optimal dose response [152]. It was estimated that about 25% of all prescribed drugs in the USA contain PGx labelling [136,153], for example, the anti-coagulant Warfarin with PGx indications of CYP2C9 genotype specific dosing [136,154]. The FDA has published a table of PGx biomarkers in drug labelling of about 135 therapeutically relevant drugs in the market [26,155].

Though the advancing technologies in genomics and bioinformatics rapidly steer PGx towards personalized therapy for patients in clinics, PGx tests are currently limited to evaluate the safety and therapeutic efficacy of drugs for thromboembolic complications associated with atrial fibrillation and/or cardiac valve replacement, human immunodeficiency virus, chronic myeloid leukaemia and gastrointestinal stromal tumours, ALL, colon, breast and lung cancers [156].

At present, the key challenges of personalized medicine include; achieving the goal of $1000 genome [157], increasing the speed of genome or exome sequencing by yet to be developed technologies [156], and making these platforms user friendly. Moreover, establishing dynamic PGx tools and databases [158-160], training physicians to apply PGx results to formulate preventive and personalized healthcare [161], and establishing privacy rules will be required to further the translational science [122,156]. At CEGMR, we are currently in the process of establishing custom-built PGx tools and databases as well as evaluating the PGx and toxicogenomics of several drugs used for the treatment of diseases afflicting Saudi population. Last, but perhaps most important, will be to, establish proper allele frequencies in populations from Saudi regions and others from across the globe [123,156].

## Discussion and summary

All of the investments in equipment in genomics infrastructure, and informatics at CEGMR aid in generating fundamental knowledge to facilitate effective decision making for individuals and families. Our primary objective is to generate the highest quality genomic data from patients and relevant family and population controls to enable opportunity for point-of-care, as well as iterative decision making based on the comparative medical data available. The advantage in the Saudi system is centralized medical care and shared experiences in families, which will also enable genome-based discoveries and treatments among families, physicians, and researchers [162].

We also recognize the importance of thoughtful deliberation on issues of societal awareness about consanguinity, infertility, and stigma associated with some genetic disorders and malformations. Therefore, the authority has launched several education and outreach programs in hospitals and schools [163]. CEGMR will continue to launch proactive communication plans with stakeholders such as government, media, children with disabilities, charitable associations, and other society representatives. Coupled with research, will also be newer ethical consenting processes that are particularly conscience of the vast array of genetic findings (and their implications) that arise from genome scanning [164]. Much of the data will be amenable to confidentiality, protection and control using an informatics-based approach [165,166]. However, there remains an urgent need in Saudi Arabia for up to date policies that comply with international standards, which implement strict control and protection of privacy and public release of bio-specimens and data [166-168]. CEGMR will also continue to develop and implement new algorithms and databases enabling iterative dynamic interpretations of data in personalized health decision making [169-171].

Other considerations relevant to translational medicine in the Saudi system include making reproductive health and ART more accessible and affordable for the average Saudi citizen so this approach can further ease the current staggering impact congenital malformations and genetic disease are having on the health care system [41]. We believe that there is a need for a strategic plan to recommend the PGD for patients with hereditary genetic disorders and receiving IVF services in the Kingdom. There also needs to be proactive and clear policies on the use of the human Embryonic Stem Cells (hESCs) and these should be easily accessed by all who are interested in the human stem cells research.

## List of abbreviations

ADRs: Adverse Drug Reactions; ALL: Acute Lymphoblastic Leukaemia; APS: Advanced Premarital Screening; ART: Assisted Reproductive Technologies; ASR: Average Standardized Rates; CDH1: Cadherin-1; CEGMR: Centre of Excellence in Genomic Medicine Research; CGH: Comparative Genomic Hybridization; CHD: Congenital Heart Defects; CIMP: CpG Island Methylator Phenotype; CIPM: Centre of Innovation for Personalized Medicine; CNS: Central Nervous System; CTGA: Catalogue of Transmission Genetics in Arabs; DMD: Duchene Muscular Dystrophy; DME: Drug Metabolizing Enzyme; EMs: Extensive Metabolizer; eSET: elective Single Embryo Transfer; ESR1: Estrogen Receptor-1; FDA: Food and Drug Administration; G1: Gastrointestinal; GCC: Gulf Cooperation Council; G6PD: Glucose-6-phosphate dehydrogenase; GWAS: Genome Wide Association Studies; HESCs: human Embryonic Stem Cells; IARC: International Agency for Research on Cancer; IMs: Intermediate Metabolizers; IVF*: in vitro* fertilization; KACST: King Abdullah City for Science and Technology; KAU: King Abdulaziz University; KFSH & RC: King Faisal Specialist Hospital & Research Centre; KSA: Kingdom of Saudi Arabia; MOH: Ministry Of Health; ND: Not Determined; NGS: Next Generation Sequencing; NHL: Non-Hodgkin`s Lymphoma; NSAIDs: Non-Steroidal Anti-Inflammatory Drugs; PGD: Pre-implantation Genetic Diagnosis; PGt: Pharmacogenetics; PGx: Pharmacogenomics; PMs: Poor Metabolizers; SADRs: Serious Adverse Drug Reactions; SCD: Sickle Cell Disease; SHGP: Saudi Human Genome Program; SNPs: Single Nucleotide Polymorphisms; T2DM: Type 2 Diabetes Mellitus; UMs: Ultra-rapid Metabolizers

## Authors’ contribution

M.A.E., S.W.S., M.A. and M.Q. developed the topic and designed the review. All authors contributed to writing up the review. M.A.E. led the teamwork. M.A., H.S., P.P. and M.A.E. designed and made the figures. S.W. S. and M.A.E. edited the manuscript.

## Disclosures

The authors disclose no competing financial interests.

## Declarations

Publication charges for this article were funded by the Center of Excellence in Genomic Medicine Research (CEGMR), King Abdulaziz University, Jeddah, 21589, Kingdom of Saudi Arabia.

This article has been published as part of *BMC Medical Genomics* Volume 8 Supplement 1, 2015: Selected articles from the 2nd International Genomic Medical Conference (IGMC 2013): Medical Genomics. The full contents of the supplement are available online at http://www.biomedcentral.com/bmcmedgenomics/supplements/8/S1
